# Supplementary material for: Variation in Fungal Community in Grapevine (Vitis vinifera) Nursery Stock Depends on Nursery, Variety and Rootstock
Source: J Fungi (Basel). 2022 Jan 3;8(1):47. doi: 10.3390/jof8010047 (PMC8778211; doi:10.3390/jof8010047)
Supplement: Supplementary file 1 [file jof-08-00047-s001.zip › jof-1507331-supplementary.pdf]

Supplementary Materials

Table S1. % GTDs (per OTUs) in graft unions as influenced by variety under nursery conditions in Catalonia, NE Spain. Results are grouped by homogenous group (determined from LS Means) and compared with previous groupings based on severity of grapevine trunk disease (GTD) symptoms related to actual disease presence in varieties under field conditions in La Mancha Designation of Origin (DO), Central Spain.

Group	Variety	LS Means	Homogenous group	Field Conditions*	Susceptibility
1	Carignan (tinta)	0.894	A		
2	Malvasia	0.672	AB	CD	High
3	Sumoll	0.601	B		
4	Autumn Royal	0.509	BC		
5	Xarel.lo vermell	0.418	BCD		
6	Tempranillo	0.356	BCDE	CD	
7	Chardonnay	0.356	CD	D	
	Macabeu	0.351	CD	CD	
	Garnacha tinta	0.343	CD	D	
	Xarel.lo	0.318	CD		
8	Merlot	0.323	CDE	D	
9	Parellada	0.283	DE	D	
	Syrah	0.265	DE	BCD	
	Pinot Noir	0.233	DE	D	
	Caladoc	0.202	DE		
10	Cabernet Sauvignon	0.209	E	BCD	Low

* Reference Field Conditions data from Chacón-Vozmediano et al., 2021 [47].

Table S2. List of GTD-related fungi. All species that appeared in this study and that have been considered as GTD-related fungus, or that are of the same genus. Method of identification was either via isolation and sequencing the amplified region ITS 1-4 (Iso), or by metabarcoding and sequencing (MBC). All fungi belong to the Phylum *Ascomycota*.

GTD Species	Method	Family	# OTUs	Tissue	Life mode	Disease caused	References
Acremonium alternatum	MBC	*Bionectriaceae*	5	Both	E		47, 48
Acremonium hansfordii	MBC	*Bionectriaceae*	1	Root			49
Acremonium sp.	MBC	*Bionectriaceae*	12	Both	P,E	'Hoja de malvon'	33, 50-55, 147
Botryosphaeria dothidea	MBC	*Botryosphaeriaceae*	2	Both	P,E,S	Botyrosphaeria dieback, Macrophoma rot	17,18, 33, 51, 52, 56-72
Botryosphaeria sp.	MBC/Iso	*Botryosphaeriaceae*	2	Both/Graft	P,E	Botyrosphaeria dieback, Macrophoma rot	51, 61, 73-76
Cadophora luteo-olivacea	MBC	*Ploettnerulaceae*	7	Both	P	Petri disease	2, 65, 72, 77, 78
Cadophora malorum	MBC	*Ploettnerulaceae*	1	Both		Soft rot	79, 80
Campylocarpon fasciculare	MBC	*Nectriaceae*	2	Both	P	Wood canker, Black foot disease	2, 51, 81-86
Campylocarpon sp.	MBC	*Nectriaceae*	1	Both			87-89
Cylindrocarpon sp.	MBC	*Nectriaceae*	7	Both	P,S	Black foot	33, 51,74, 75, 77, 90, 91
Dactylonectria alcacerensis	Iso	*Nectriaceae*		Root	P	Black foot	86, 92
Dactylonectria anthuriicola	Iso	*Nectriaceae*		Root			88
Dactylonectria sp.	Iso	*Nectriaceae*		Both	P	Black foot	88, 89
Diaporthe celeris	Iso	*Diaporthaceae*		Graft	P	Canker and Arm swelling	93

Diaporthe foeniculina	MBC	*Diaporthaceae*	1	Both	P	Canker and Arm swelling	18, 94-97
Diaporthe hispaniae	Iso	*Diaporthaceae*		Graft			93
Diaporthe rudis	MBC	*Diaporthaceae*	1	Both	P,S	Canker	33, 64, 95, 96, 98-100
Diaporthe sp.	MBC/Iso	*Diaporthaceae*	1	Both/Both	P	Canker	18, 77, 94, 101-105
Diplodia sp.	MBC/Iso	*Botryosphaeriaceae*	4	Both/Both	P	Canker	77, 104, 106, 107
Eucasphaeria capensis	MBC	*Niessliaceae*	1	Both	P	Eutypa dieback	72
Ilyonectria liriodendri	Iso	*Nectriaceae*		Root	P	Black foot	51, 82, 85, 92, 108, 109
Ilyonectria sp.	Iso	*Nectriaceae*		Both	P,S	Black foot	33, 85, 108, 110
Lasioplodia sp.	MBC					Botryosphaeria die back	111
Nectria sp.	MBC	*Nectriaceae*	4	Both	S		112, 113
Neofusicoccum parvum	MBC/Iso	*Botryosphaeriaceae*	2	Both/Graft	P,E,S	Botryosphaeria die back	33, 52, 54, 60, 65, 83, 114-121, 147
Neofusicoccum sp.	MBC/Iso	*Botryosphaeriaceae*	2	Both/Both			107
Paraphoma sp.	MBC	*Phaeosphaeriaceae*	1	Both			
Pestalotiopsis sp.	MBC/Iso	*Sporocadaceae*	2	Both/Graft	P,E,S	Fruit rot	33, 51, 64, 122
Phaeoacremonium minimum	MBC	*Togniniaceae*	3	Both	P	Esca	64, 85, 108, 123, 124
Phaeoacremonium parasiticum	MBC	*Togniniaceae*	1	Both	P	Esca	50, 125, 126
Phaeoacremonium sp.	MBC	*Togniniaceae*	7	Both	P,S	Esca	33, 50, 53, 127, 128
Phaeomoniella chlamydospora	MBC	*Phaeomoniellaceae*	3	Both	P,E	Esca	129-136
Phaeomoniella sp.	MBC	*Phaeomoniellaceae*	2	Both		Esca	128
Phoma sp.	MBC	*Didymellaceae*	8	Both	P,E,S	Leaf and stem lesions	33, 54, 57, 77, 147
Phomopsis ampelina	MBC	*Diaporthaceae*	1	Both	P, S	Phomopsis cane and leaf spot/Deadarm/Phomopsis dieback	18, 57
Phomopsis sp.	MBC	*Diaporthaceae*	5	Both	P,E,S	Phomopsis cane and leaf spot/Deadarm/Phomopsis dieback	57
Pleurostoma richardsiae	MBC	*Pleurostomataceae*	1	Graft	P	Trunk disease	10, 71, 128
Thelonectria sp.	Iso	*Nectriaceae*		Root	P	Black foot disease	65
Truncatella angustata	MBC/Iso	*Sporocadaceae*	1	Root/Both	P,E		77, 128, 137, 138, 147, 150
Truncatella sp.	MBC	*Sporocadaceae*	1	Both			150

Table S3. Relative abundance (%) of GTD-related OTUs (species) in each tissue type. Abundances are calculated considering the totals for each tissue separately.

OTU	Graft union	Root collar
Acremonium alternaturm	11.52%	0.33%
Botryosphaeria dothidea	0.55%	0.11%
Cadophora luteo-olivacea	30.44%	4.61%
Campylocarpon fasciculare	-	0.15%
Cylindrocarpon sp.	0.01%	0.13%
Diplodia sp.	0.55%	0.11%
Neofusicoccum parvum	1.25%	0.04%
Phaeoacremonium minimum	1.28%	0.44%
Phaeomoniella chlamydospora	2.09%	1.21%
Phomopsis ampelina	0.69%	0.02%
Phomopsis sp.	0.21%	0.01%
**Total**	48.6%	7.2%

Table S4. Relative abundance (%) of GTD-related isolates (species) in each tissue type. Abundances are calculated considering the totals for each tissue separately.

Isolate	Graft union	Root collar
Botryosphaeria sp.	3.39%	-
Dactylonectria alcacerensis	-	4.18%
Dactylonectria anthuriicola	-	0.52%
Dactylonectria sp.	0.26%	2.35%
Diaporthe celeris	0.78%	-
Diaporthe hispaniae	1.31%	-
Diaporthe sp.	1.04%	0.52%
Diplodia sp.	3.13.%	0.78%
Ilyonectria liriodendra	-	0.78%
Ilyonectria sp.	0.26%	5.22%
Neofusicoccum parvum	1.31%	-
Neofusicoccum sp.	6.01%	1.31%
Pestalotiopsis sp.	0.26%	-
Thelonectria sp.	-	2.09%
Truncatella angustata	0.52%	0.52%
**Total**	18.28%	18.28%

Table S5. Indicator species in **(A)** graft unions and **(B)** root collars. Graft unions were analyzed by variety and root crowns by rootstock. For each species (OTU), we indicate the community habitat that had the highest correlation with the species. Correlation values (stat) and the statistical significance of the correlation (p-values) are reported. GTD-related indicator species are marked in bold, biocontrol species are marked with an asterisk. Annotation next to the numbers indicates significance level: *** < 0.001, ** < 0.01, * < 0.05, n.s. = not significant (> 0.05).

A. Graft unions

OTU	Community habitat	stat	p-value
Coniochaeta sp.	Autumn Royal	0.528	0.0214 *
Robillarda sessilis	Caladoc	0.408	0.0486 *
unknown_42	Caladoc	0.833	0.0007 ***
Coprinellus sp.	Carignan	0.446	0.0262 *
Diplodia sp.	Carignan	0.698	0.0032 **
Lasiodiplodia sp.	Carignan	0.572	0.0084 **
Phaeoacremonium sp.	Carignan	0.502	0.0333 *
Phaeomoniella chlamydospora	Carignan	0.729	0.0013 **
Thelebolus ellipsoideus	Carignan	0.444	0.0196 *
unknown_1218	Carignan	0.447	0.0226 *
unknown_Ascomycota_94	Carignan	0.447	0.0125 *
unknown_382	Carignan	0.427	0.0427 *
Erysiphe sp.	Malvasia	0.546	0.0123 *

Neofusicoccum sp._391	Malvasia	0.549	0.014 *
Neofusicoccum sp._398	Malvasia	0.458	0.0443 *
unknown_604	Malvasia	0.577	0.008 **
unknown_Basidiomycota_281	Malvasia	0.569	0.0086 **
unknown_1250	Malvasia	0.566	0.0048 **
unknown_1230	Malvasia	0.456	0.0336 *
Septoria sp.	Pinot Noir	0.55	0.014 *
unknown_Ascomycota_141	Pinot Noir	0.469	0.0282 *
Acremonium alternatum	Sumoll	0.396	0.0091 **
Pyrenophora dictyoides	Sumoll	0.406	0.0391 *
unknown_554	Sumoll	0.528	0.0274 *
unknown_683	Sumoll	0.485	0.0337 *
unknown_1582	Sumoll	0.479	0.0228 *
Cadophora malorum	Tempranillo	0.499	0.0234 *
Leptosphaeria sp.	Tempranillo	0.544	0.0106 *
unknown_Ascomycota_12	Tempranillo	0.595	0.019 *
unknown_Ascomycota_303	Tempranillo	0.53	0.013 *
unknown_1559	Tempranillo	0.459	0.033 *
Mortierella hyalina	Xarel.lo vermell	0.544	0.0143 *

B. Root collars

OTU	Community habitat	stat	p-value
Fusarium sp.	R-110	0.628	0.0112 *
Acremonium sp._1286	41B	0.272	0.0218 *
Acremonium sp._812	41B	0.199	0.0493 *
Cercophora sp.	41B	0.201	0.0233 *
Circinotrichum maculiforme	41B	0.529	0.0143 *
Coprinellus radians	41B	0.204	0.0497 *
Cryptococcus sp.	41B	0.271	0.0031 **
Exidia sp.	41B	0.277	0.0225 *
Harposporium sp.	41B	0.293	0.01 **
Lasiodiplodia sp.	41B	0.199	0.0274 *
Leptosphaeria sp.	41B	0.203	0.0317 *
*Lophiostoma sp._839 **	41B	0.391	0.0046 **
*Lophiostoma sp._1100 **	41B	0.231	0.0497 *
*Lophiostoma sp._390 **	41B	0.191	0.0396 *
Massarina sp.	41B	0.286	0.0078 **
Mortierella hyalina	41B	0.281	0.0048 **
Radulidium subulatum	41B	0.19	0.0297 *
Seimatosporium sp.	41B	0.204	0.0276 *
Talaromyces amestolkiae	41B	0.241	0.0221 *
Tilletiopsis washingtonensis	41B	0.266	0.0206 *
Truncatella sp.	41B	0.254	0.0158 *

unknown_Ascomycota_99	41B	0.378	0.017 *
unknown_Basidiomycota_219	41B	0.368	0.0202 *
unknown_1174	41B	0.319	0.0032 **
unknown_Ascomycota_925	41B	0.283	0.0131 *
unknown_360	41B	0.275	0.0069 **
unknown_Ascomycota_581	41B	0.262	0.0465 *
unknown_666	41B	0.253	0.0208 *
unknown_Ascomycota_189	41B	0.204	0.0451 *
Meira nashicola	RU-140	0.225	0.0359 *
Thanatephorus cucumeris	RU-140	0.386	0.0286 *
unknown_21	RU-140	0.517	0.0465 *
*Alternaria sp. **	SO4	0.303	0.0268 *
Colletotrichum sp.	SO4	0.299	0.0484 *
Oidiodendron cereale	SO4	0.258	0.0232 *
Podospora sp.	SO4	0.264	0.0426 *
Sarocladium kiliense	SO4	0.355	0.0472 *
unknown_Ascomycota_48	SO4	0.318	0.0464 *
unknown_771	SO4	0.266	0.0333 *
unknown_Basidiomycota_188	SO4	0.248	0.0473 *
